# Isolation, Identification, and Characterization of Novel Environmental Bacteria with Polyurethane-Degrading Activity

**DOI:** 10.3390/biology14091307

**Published:** 2025-09-22

**Authors:** Marta Muñoz-Martí, Virtudes Navarro Bañón, Mª Carmen García-Poyo, Carlos Castaño Forte, Josefina Garrido, Jose María Orts, Andrea Huguet, Jorge García-Hernández, María Ángeles Castillo

**Affiliations:** 1 Materials, Adhesion and Polymers Area, R&D Department, Technology Centre of Furniture and Wood of the Region of Murcia (CETEM), 30510 Yecla, Spain; v.navarro@cetem.es (V.N.B.); mc.garcia@cetem.es (M.C.G.-P.); c.castano@cetem.es (C.C.F.); josefina.garrido@cetem.es (J.G.); 2 Advanced Centre of Applied Microbiology (CAMA), Universitat Politècnica de València (UPV), 46022 Valencia, Spain; andreaupvbiotec@gmail.com (A.H.); jorgarhe@btc.upv.es (J.G.-H.); mcastill@btc.upv.es (M.Á.C.); 3 Department of Biochemistry and Molecular Biology, Facultad de Farmacia, University of Seville, 41012 Seville, Spain; jorts1@us.es

**Keywords:** polyurethane, biodegradation, environmental bacteria, bacterial isolation, enzymatic activity, Impranil, bioremediation

## Abstract

Polyurethane (PU) is a widely used plastic material whose persistence in the environment entails a serious ecological challenge. This study aimed to isolate and characterize environmental bacteria capable of degrading PU, using Impranil DLN as a model substrate, and to investigate their enzymatic mechanisms and phylogenetic relationships. A total of 31 bacterial isolates were obtained from four waste accumulation sites and taxonomically identified across 12 different genera using MALDI-TOF MS and 16S rRNA gene sequencing. This analysis revealed genera not previously reported as PU biodegraders, including *Priesta*, *Dermacoccus*, *Gordonia*, *Micrococcus*, *Pseudarthrobacter*, and *Agromyces*. The *Bacillus cereus* clade was the most prevalent group, followed by the *Priestia megaterium* clade and *Achromobacter* sp. Biodegradation assays revealed high variability among strains, with the most efficient degrading over 90% of Impranil DLN. Protease activity was the most frequently detected enzymatic function, followed by urease and esterase activities. However, no clear correlation was observed between enzymatic profiles and degradation efficiency. Selected strains were tested on polyether PU foam, revealing biodegradative activity, an uncommon observation for bacteria on such recalcitrant material. These findings contribute to our understanding of bacterial diversity and enzymatic mechanisms involved in PU biodegradation, as well as their potential applications in plastic waste bioremediation.

## Simple Summary

Plastic pollution is a major environmental problem, and polyurethane is a particularly challenging type of plastic to break down. In this study, we searched waste sites for bacteria that can naturally degrade polyurethane. By using a simple polyurethane model material to test their abilities, we identified 31 different bacteria belonging to 12 groups, including some that had never previously been associated with breaking down this type of plastic. The most prevalent bacteria belonged to the *Bacillus* group, followed by the *Priestia* and the *Achromobacter* species, and some bacteria were able to degrade over 90% of the test material. We also studied the enzymes that these bacteria use and found that proteases were the most common, followed by ureases and esterases. However, there was no simple link between the type of enzymes and how effectively the bacteria degraded the plastic. Lastly, a few selected bacteria were tested on polyurethane foam, which is a more resistant material, and the ability to break it down was demonstrated. These results help us to understand which bacteria can attack polyurethane and how they do it. They also suggest new ways in which bacteria could be used to reduce plastic pollution in the environment.

## 1. Introduction

Plastics play an indispensable role in contemporary human life due to their unique properties, including strength, durability, and low production costs [1]. These characteristics make them ideal for a variety of industrial applications and everyday uses. However, irresponsible production and consumption practices have led to severe environmental consequences [1,2]. Despite advances in recycling methods, some plastics still lack sustainable disposal solutions, with polyurethane (PU) being a good example [3,4].

PU is the sixth most widely used polymer in the world, with a global market volume of around 25.8 million metric tons in 2022 and a projected increase to 31.3 million tons by 2030 [5]. The great diversity of PU properties makes it versatile for synthesis in a wide range of products, including foams, adhesives, elastomers, and coatings [6]. In addition, PU’s high resistance to environmental degradation makes it an ideal material for long-term applications. However, while PU’s high resistance to environmental degradation is advantageous for long-term applications, it also results in low recyclability and contributes to the accumulation of persistent PU waste. This accumulation has been linked to carbon resource loss and the disruption of ecosystems, exacerbating global environmental issues [7,8]. Several industrial strategies for PU recycling have already been established, although they are still subject to optimization. Physical recycling results in low-quality and low-value products. Conversely, chemical recycling entails a significant environmental impact due to the use of toxic solvents and extreme operating conditions [8]. Therefore, alternative and complementary strategies to address PU pollution have emerged as a pressing global concern, with biodegradation and biological recycling being considered since the late 20th century due to its green, non-toxic, and sustainable nature [7,9,10]. Although biological PU recycling strategies remain limited to the laboratory scale, significant advances have already been achieved in this field, highlighting their potential as complementary approaches to existing recycling technologies. Several species of fungi and bacteria have been reported to metabolize PU through enzymatic hydrolysis, a process that causes the cleavage of the polymer and the release of low-molecular-weight compounds [7,11,12]. Thus, if the enzymatic hydrolysis can be successfully replicated in the laboratory, the resulting molecules can be used as raw materials for synthesizing new polymers [13]. Subsequently, various enzymes involved in PU depolymerization—esterases, ureases, amidases, proteases, cutinases, and oxidoreductases—have been progressively identified [7]. Moreover, the susceptibility of PU to biodegradation is strongly influenced by its chemical composition, macromolecular architecture, bond types, crystallinity, and molar mass [7,14]. For instance, polyester PU is more susceptible to microbial biodegradation than polyether PU. Additionally, aqueous dispersions and films are easier (more vulnerable) to attack than foamed materials [4,7,15].

Therefore, developing new biotechnological tools for PU biodegradation is important, particularly for highly recalcitrant polyether PU and foams. Some strategies focus on isolating microorganisms and enzymes with a high potential for PU degradation. Among bacteria, notable genera include *Comamonas* sp. [11], *Bacillus* sp. [8,16,17,18], *Pseudomonas* sp. [16,19], *Serratia* sp. [20], *Streptomyces* [21], and *Moraxella* [22]. However, only the strains reported by Ji et al. (2024) [18] and Zeng et al. (2024) [23] have demonstrated activity against PU foams. Although some fungi have been reported to degrade PU foams [24,25], most bacteria exhibit a limited degradation capacity toward commercial PU materials, and reports of bacterial strains with a validated efficiency are scarce.

In this context, the present study aimed to isolate and identify novel environmental bacteria with PU-degrading capabilities, assess their biodegradation potential on various PU substrates, and characterize the enzymatic activities involved in the degradation process.

## 2. Materials and Methods

### 2.1. Culture Media

A mineral saline medium (MM) was prepared based on the formulation provided by Oceguera-Cervantes et al. (2007) [26], with the following composition (g/L): KH_2_PO_4_, 2.0; K_2_HPO_4_, 7.0; NH_4_NO_3_, 1.0; MgSO_4_ × 7H_2_O, 0.1; ZnSO_4_ × 7H_2_O, 0.001; CuSO_4_ × 5H_2_O, 1 × 10^4^; FeSO_4_ × 7H_2_O, 0.01; and MnSO_4_ × 1H_2_O, 0.002. All salts were purchased from Labkem (Premià de Dalt, Spain). 2 g/L of water-based polyester PU resin Impranil DLN W50 (DLN) from Bayer MaterialScience AG (Leverkusen, Germany) was added after MM autoclaving to create a minimal medium with PU as the only carbon source: MM-PU. In the case of a solid medium, 15 g/L agar (Liofilchem, Roseto degli Abruzzi, Italy) was added before sterilization. Nutrient broth (NB) (Liofilchem, Roseto degli Abruzzi, Italy) was used for bacterial growth and nutrient agar (Liofilchem, Roseto degli Abruzzi, Italy) supplemented with 40 g/L glucose (Labkem, Premià de Dalt, Spain) (MEA) was used for pure culture establishment and maintenance.

### 2.2. Polyurethane Foams Synthesis

Polyurethane foams (FPU) were synthesized in the laboratory using a 50:50 polyol blend composed of the glycerol-initiated triol heteropolymer polyether Voranol™ 8322 (DOW Chemical Company, Midland, MI, USA) and the corn oil-based polyether biopolyol Ecotrion H 1000 (Gantrade Europe Ltd., Montvale, NJ, USA). The formulation included 3.5 parts per hundred polyols (pph) of distilled water, 1.1 pph of silicone-based surfactant TEGOSTAB B8291^®^ (Evonik, Essen, Germany), and 0.14 pph of the tertiary amine catalyst TEGOAMIN^®^ B (Evonik, Essen, Germany). All components were weighed using a precision balance (PM2500, Mettler, Hong Kong, China) and mixed for 90 s at 2000 rpm using a mechanical stirrer (IKA RW20, Barcelona, Spain).

Subsequently, 0.2 pph of tin-based catalyst Kosmos T9^®^ (Evonik, Essen, Germany) was added and mixed for an additional 30 s under the same stirring conditions. Finally, 2,4- and 2,6-toluene diisocyanate isomers (80:20 ratio), Voranate T-80™ (DOW Chemical Company, Midland, MI, USA), were added with an isocyanate index of 115. The mixture was stirred for 8 s to ensure complete homogenization, then poured into a mold and cured at room temperature for 72 h.

### 2.3. Isolation of PU Biodegrading Bacteria

Environmental samples were collected from four waste accumulation areas, selected for their varied histories of PU accumulation and microbial exposure, with the aim of obtaining broad microbial diversity. These included the PU foam by-product disposal area in an upholstery factory (TM1), the PU foam by-product disposal area in a mattress factory (TM2), the surrounding area of a former upholstery factory (TM3), and a local landfill (TM4).

Three subsamples of soil, vegetal material, and waste, preferably PU foams, were collected at three different points in the same area. Once in the laboratory, these subsamples were milled to 2 mm in an ultracentrifugal mill (Retsch ZM 300, Haan, Germany). The subsamples were then joined into a single homogeneous sample for each area, which was weighed and equally distributed into flasks containing MM (controls) and MM-PU broth at a 1:10 sample:media (*w*/*v*) ratio, thereby normalizing the samples moisture.

The samples were previously dried to the flasks, which were incubated in a rotary shaker (LBX Instruments, Premià de Dalt, Barcelona, Spain) for 7 days at 30 °C. Following incubation, aliquots from each flask were plated onto MM (control) and MM-PU agar and incubated at 30 °C until visible growth occurred. The selection of DLN biodegrading bacteria was based on the fact that DLN becomes transparent from milky opaque when degraded. Colonies exhibiting a clearing halo in MM-PU were subcultured in MEA to establish pure cultures of PU biodegrading isolates and preserved in a freezer suspended in 30% glycerol solution (Labkem, Premià de Dalt, Spain). This method was adapted and modified from Álvarez-Barragán et al. (2016) [24].

### 2.4. Identification of Isolated Bacteria

All the isolates were analyzed by matrix-assisted laser desorption/ionization time-of-flight mass spectrometry (MALDI-TOF MS) using a Microflex L20 (Bruker Daltonics, Fremont, CA, USA) equipped with an N2 laser. According to the protocol recommended by Bruker Daltonics (http://www.bdal.de, accessed on 16 April 2025), it would be achieved by using the ‘extended direct transfer’ method.

Spectra were acquired in positive linear ion mode with an acceleration voltage of 20 kV. Each spectrum corresponded to the sum of 240 shots per target, the mass range used for the analysis was 2000–20,000 Da, and 3 spectra per strain were obtained. Identification was performed using the MALDI Biotyper Realtime Classification method concerning the MBT 7854 (Bruker Daltonics) and the MBT 7311_RUO (Bruker Daltonics) databases and the MALDI-TOF database of the Spanish Type Culture Collection (CECT) and the DWL (Drinking Water Library). Data were processed with BioTyper 3.1 (Bruker Daltonics) as described by Maier et al. (2006) [27].

Strains that MALDI-TOF MS could not be identify were analyzed by partial 16S rRNA gene sequencing, as described in Arahal et al. (2008) [28]. The DNA was amplified by PCR in a thermocycler using universal primers 616V: 5′-AGA GTT TGA TYM TGG CTC AG -3′ and 699R: 5′-RGG GTT GCG CTC GTT -3′ [28], encompassing hypervariable regions V1–V6. PCR products were checked by electrophoresis on agarose gel 1% (*w*/*v*) in Tris-Borate-EDTA buffer at 135 V for 25 min. PCR fragments were sequenced on an ABI 3730xl sequencer (Applied Biosystems, Waltham, MA, USA) using the same PCR primers at a concentration of 5 nM. The 16S rRNA gene sequence was analyzed on BLAST+ 2.16.0 [29].

### 2.5. Quantitative DLN Biodegradation Analysis

Overnight NB cultures of each isolate were harvested by centrifugation at 4000 rpm for 10 min, washed with sterile water, and resuspended in MM to obtain a bacterial cell suspension with OD600 of 1. In addition, serial dilutions were plated in MEA to check the cell concentration. One of the suspensions was inoculated in MM-PU broth tubes at 25% (*v*/*v*) in triplicate. One tube was inoculated with distilled water as a negative control. After incubating for 14 days at 30 °C, cultures were filtrated by 0.4 µm acetate filters (Labbox, Premià de Dalt, Barcelona, Spain). Filtrate turbidity was measured in a spectrophotometer (ONDA V-10 PLUS, Lbx Instruments, Premià de Dalt, Barcelona, Spain) at 600 nm in triplicate using MM as the blank. DLN biodegradation was determined using a standard linear (r^2^ ≈ 0.99) curve generated with DLN dilutions from 2g/l (1/2, 1/3, 1/4, 1/6, 1/8, and 1/12). This method was adapted and modified from Álvarez-Barragán et al. (2016) [24].

### 2.6. Enzymatic Analysis

The polyurethane-degrading capability has been associated primarily with three enzymatic activities, as described by Oceguera-Cervantes et al. (2007) [26].

Protease activity was determined by the method described by Howard and Blake (1998) [30]. Strains were subcultured on YES agar medium (composition in g/L: K_2_HPO_4_, 1.0; KH_2_PO_4_, 0.5; MgSO_4_·7H_2_O, 0.5; MnCl_2_·4H_2_O, 0.001; CuCl_2_·2H_2_O, 1.4 × 10^−5^; ZnCl_2_, 1.1 × 10^−5^; CoCl_2_·6H_2_O, 2 × 10^−5^; Na_2_MoO_4_·2H_2_O, 1.3 × 10^−5^; FeCl_3_·6H_2_O, 7.5 × 10^−5^; agar, 15; gelatin, 0.02; and pH 7.0). Plates were incubated at 37 °C for 48 h, followed by staining with 0.1% Coomassie Brilliant Blue R-250 in acetic acid: methanol (3:1, *v*/*v*) for 30 min. Plates were then washed with the same acetic acid: methanol solution. Proteolytic activity was indicated by the presence of a clear, unstained halo surrounding the bacterial colonies. Urease activity was evaluated according to the method of Christensen (1946) [31], using Christensen’s urea agar (composition in g/L: urea, 20; agar, 15; NaCl, 5; KH_2_PO_4_, 2; peptone, 1; glucose, 1; and phenol red, 0.012). After incubation at 37 °C for 48 h, a color change from yellow to pink was observed in colonies exhibiting urease activity, indicating an alkaline pH shift due to ammonia production during urea hydrolysis [24].

On the other hand, esterase activity was evaluated using the method described by Sierra (1957) [32]. Strains were subcultured on Tween 80 agar (composition in g/L: peptone, 10; NaCl, 5; CaCl_2_, 0.1; agar, 12; and supplemented with Tween 80, 10 mL/L) and incubated at 37 °C for 120 h. Esterase-positive strains showed a white precipitate halo surrounding the colonies due to calcium salt formation. Pseudomonas aeruginosa was used as a positive control for all three enzymatic assays, as it is known to express protease, urease, and esterase activities, hereby serving as a reliable reference strain for experimental validation.

### 2.7. FPU Biodegradation Assay

Biodegradation assays were conducted with 3 × 3 × 1 cm FPU pieces that were washed with distilled water and sterilized by ultraviolet radiation and heating at 90 °C, also drying to constant weight. Phylogenetic groups previously exhibiting biodegradation rates below 70% were selected for the assay. This selection strategy aimed to represent the diversity of phylogenetic groups identified in this study and as an operational criterion to select the highest-performing isolates. Bacteria were harvested from overnight NB cultures by centrifugation (4000 rpm, 10 min). After washing, the pellet was suspended in distilled water and OD600 was adjusted to 1.0. FPU pieces were inoculated with 1 mL of cellular suspension in previously autoclaved flasks with 40 mL of NB 25% and incubated in a rotary shaker (LBX Instruments, Premià de Dalt, Barcelona, Spain) at 30 °C. Decreasing concentrations of NB medium (20, 15, and 10%) were used to refresh the culture once per week. After 1 month, FPU was sterilized, washed, and dried to constant weight with the same method used for FPU preparation. Biodegradation was calculated by weight difference and further characterized using attenuated Total Reflectance-Fourier Transformed Infrared Spectroscopy (FTIR) and scanning electron microscopy (SEM). FTIR spectra were obtained with an Alpha II spectrometer (Bruker, Karlsruhe, Germany) equipped with a monolithic diamond crystal (Platinum ATR, Bruker, Karlsruhe, Germany). Measurements were collected in transmittance mode over the spectral range of 4000–400 cm^−1^, with a resolution of 4 cm^−1^ and 24 scans per sample. Data was processed using Opus 8.2.28 software. For SEM analysis, FPU samples were cut into thin sections, sputter-coated with gold, and examined with a Hitachi S-3500N microscope (Chiyoda, Japan) operated at 10 kV. Images were acquired at magnifications of 25×.

## 3. Results

### 3.1. Direct Screening of PU Biodegrading Bacteria

**Figure 1:**
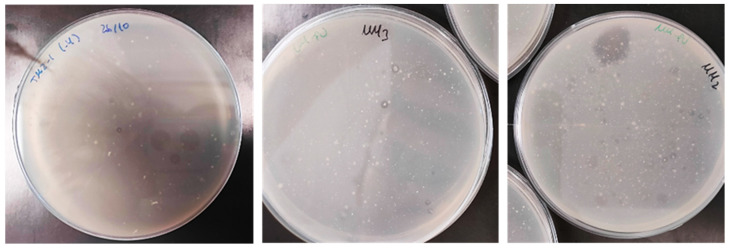
Growth and halos formed in MM-PU plates by DLN degradation by some of the bacterial isolates.

Thirty-one PU biodegrading bacterial isolates were obtained by selective growth from environmental samples on MM-PU. Colonies were selected by their halo-producing capacity in MM-PU agar, thereby demonstrating polyester PU biodegradation (Figure 1). This method, unlike that used in other works involving bacteria [20,33,34], directly obtains isolates that can grow on PU, saving the step of classical isolation followed by screening on PU. Different numbers of bacterial strains were isolated from each sampling site. The highest number was obtained from TM2 (mattress factory), yielding 19 isolates. TM4 (municipal dump) followed with 11 isolates, while TM3 (former upholstery factory) yielded two isolates. A single strain was isolated from TM1 (sofa factory).

### 3.2. Bacteria Identification

**Figure 2:**
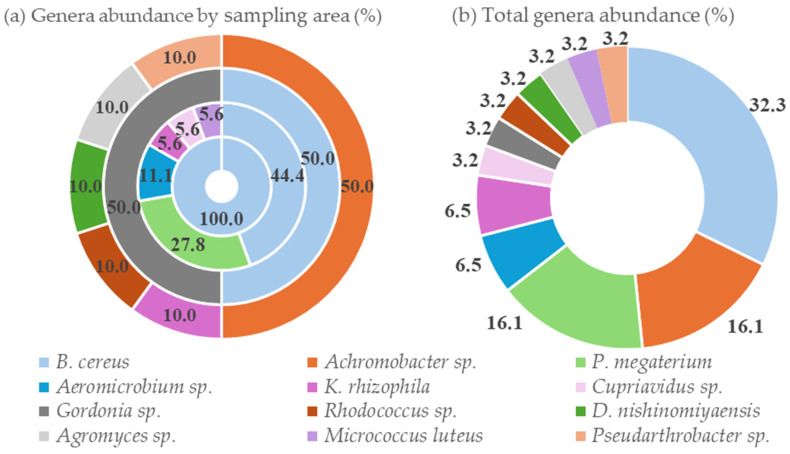
Relative abundance of bacterial genera: (**a**) Distribution by sampling area (from inner to outer rings: TM1, TM2, TM3, and TM4); (**b**) overall genera abundance across all the sampling areas.

A comprehensive approach combining MALDI-TOF MS and 16S rRNA gene sequencing was employed to identify the bacterial isolates. This dual-method strategy facilitated genus-level identification for all isolates, resulting in 12 different genera. Figure 2 illustrates the relative abundance of the identified bacterial genera, both by individual sampling area and as a total distribution across all locations. Also, the most likely species within the genus were identified in some instances.

MALDI-TOF MS analysis revealed a high probability of identification of *Kocuria rhizophila* and *Micrococcus luteus* and a probable identification of *Dermacoccus nishinomiyaensis*. Phylogenetically closely related species have very similar MALDI-TOF MS profiles and are indistinguishable, being grouped into clades [35]. Two clades were identified in this work: *Bacillus cereus* and *Priestia megaterium* clades.

Other isolates were only identified at the genus level: *Cupriavidus* sp. and *Agrobacterium* sp. This technique could not identify the remaining isolates due to its ineffectiveness on spore-forming species or the absence in the database [36]. In such cases, 16S rRNA gene sequencing was used, identifying *Rhodococcus erythropolis*.

**Figure 3:**
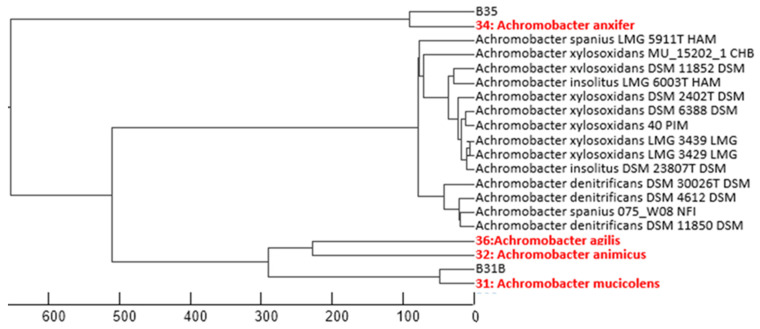
Phylogenetic dendrogram of *Achromobacter* strains isolated in this study (highlighted in red), constructed from partial 16S rRNA gene sequences. The scale bar indicates genetic distance.

Given the high similarity percentage between the 16S rRNA gene sequences of other isolates and several species of *Achromobacter* and *Pseudarthrobacter*, this identification was considered at the phylogenetic group level. Especially, *Achromobacter* isolates showed a high similarity (>99%) to multiple species within this genus. Figure 3 represents the phylogenetic relationships based on partial 16S rRNA gene sequences. Notably, three isolates within the *Aeromicrobium* and *Gordonia* genera exhibited less than 99% sequence similarity to known species, suggesting the possibility of novel species [36]. However, confirmation of their novelty would require whole-genome sequencing and comprehensive taxonomic analyses, including assessments of cell morphology, motility, biochemical profiles, and growth parameters [37,38]. This analysis will be addressed in further studies.

### 3.3. Quantitative PU Biodegradation Analysis

**Figure 4:**
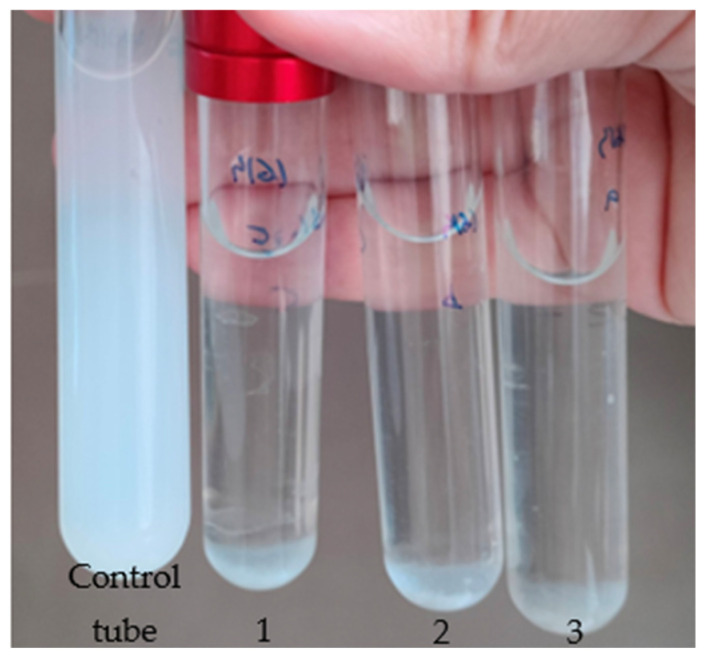
Representative result of the DLN biodegradation assay. The control tube was inoculated with sterile water, while tubes 1, 2, and 3 (triplicate) were inoculated with a bacterial isolate and incubated for four days.

**Figure 5:**
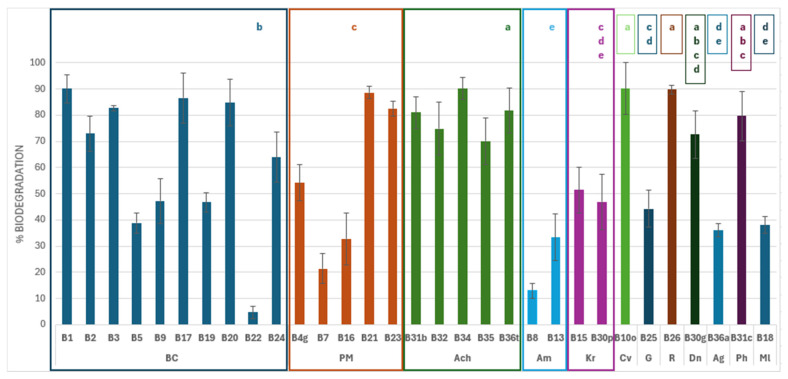
Impranil DLN W-50 (DLN) biodegradation (%) obtained with the isolates, grouped by identification: Bc, *Bacillus cereus* clade; Pm, *Priestia megaterium* clade; Ach, *Achromobacter* sp.; Am, *Aeromicrobium* sp.; Kr, *Kokuria rhizophila*; Cv, *Cupriavidus* sp.; G, *Gordonia* sp.; R, *Rhodococcus erythropolis*.; Dn, *Dermacoccus nishinomiyaensis*; Ag, *Agrobacterium* sp.; Ph, *Pseudarthrobacter* sp.; and Ml, *Micrococcus luteus*. The taxonomic groups labeled with different superscripts (a–e) showed significant differences in the average biodegradation percentages (*p* < 0.05).

A total of 100% of the 31 bacterial isolates exhibited PU biodegradation of DLN, with values ranging from 4.7 to 90.2% and with 16 isolates exhibiting values exceeding 70% biodegradation (Table 1, Figure 4). An ANOVA analysis was performed on the results to establish a relationship between the strains according to their sampling location (Table 1) or identification (Figure 5).

The highest percentage of biodegradation occurred at TM1, where the only isolated strain showed a biodegradation of 90.1%. This differs significantly (*p* < 0.05) from the results obtained for strains from TM2 and TM3. However, there were not any significant differences observed in comparison to the results obtained from TM4. Conversely, the strains isolated from TM2 were the most numerous and exhibited high variability. However, their results were significantly lower than those observed in the strains from TM1 and TM4.

Comparable degradation percentages were observed among isolates belonging to the *Achromobacter* genus (standard deviation 7.6) and *K. rhizophila* (standard deviation 3.1). Within the *Achromobacter* genus, the different strains exhibited a uniform biodegradation response, with percentages ranging from approximately 70 to 90%. These patterns suggest that they may share similar enzymatic pathways, since they have evolved together. Statistically, this clade exhibits the highest efficiency in DLN degradation, demonstrating significant differences (*p* < 0.05) compared to most other groups and outperforming *Bacillus*, *Priestia*, *Aeromicrobium*, *Kocuria*, *Gordonia*, *Micrococcus*, and *Agromyces*.

In contrast, the two *Kocuria* strains showed intermediate efficiency, with degradation percentages close to 50%. Although their activity did not differ significantly from clades with a lower degradation activity, they showed statistical differences (*p* < 0.05) compared to clades with a high biodegradation capacity, such as *Bacillus*, *Achromobacter*, *Cupriavidus*, and *Rhodococcus*. On the other hand, strains belonging to *Aeromicrobium* showed the lowest levels of biodegradation, with percentages below 35%. *Aeromicrobium* strains were significantly less efficient at degrading DLN than other genera, except for *Kocuria*, *Micrococcus*, and *Agromyces*, for which no significant differences were observed.

The clades *B. cereus* and *P. megaterium* showed a wide distribution. Two subgroups could be differentiated in *B. cereus*, one with a greater biodegradation capacity (B1, B2, B3, B17, B20, and B24), and the other with less degradation activity (B5, B9, B19, and B22). An ANOVA analysis revealed that strains belonging to the same clade exhibited significantly different levels of DLN biodegradation, ranging from 4.7% for strain B22 to 90.1% for strain B1. This wide variability suggests functional diversity within the clade, as the genus includes both highly efficient and low-activity strains. This wide range indicates that these strains have evolved to adopt different PU biodegradation mechanisms despite having a common phylogenetic origin.

A similar pattern was observed within the *Priestia* clade, where there is also significant variability in the degradative capacity. Some strains, such as B21 and B23, demonstrate capacities close to 90%, while others, such as B7 and B16, show biodegradation below 35%. Statistically, this clade exhibits a degradation pattern similar to that of *Bacillus*, with no significant differences between them.

### 3.4. Enzymatic Analysis

Protease, urease and esterase are among the most identified enzymes in microorganisms associated with PU degradation [9,26,30,39]. In the present study, the enzymatic activity of all 31 bacterial isolates was evaluated using differential media. *Pseudomonas aeruginosa* CECT 110T was used as a positive control, exhibiting moderate activity for the three enzymes.

**Figure 6:**
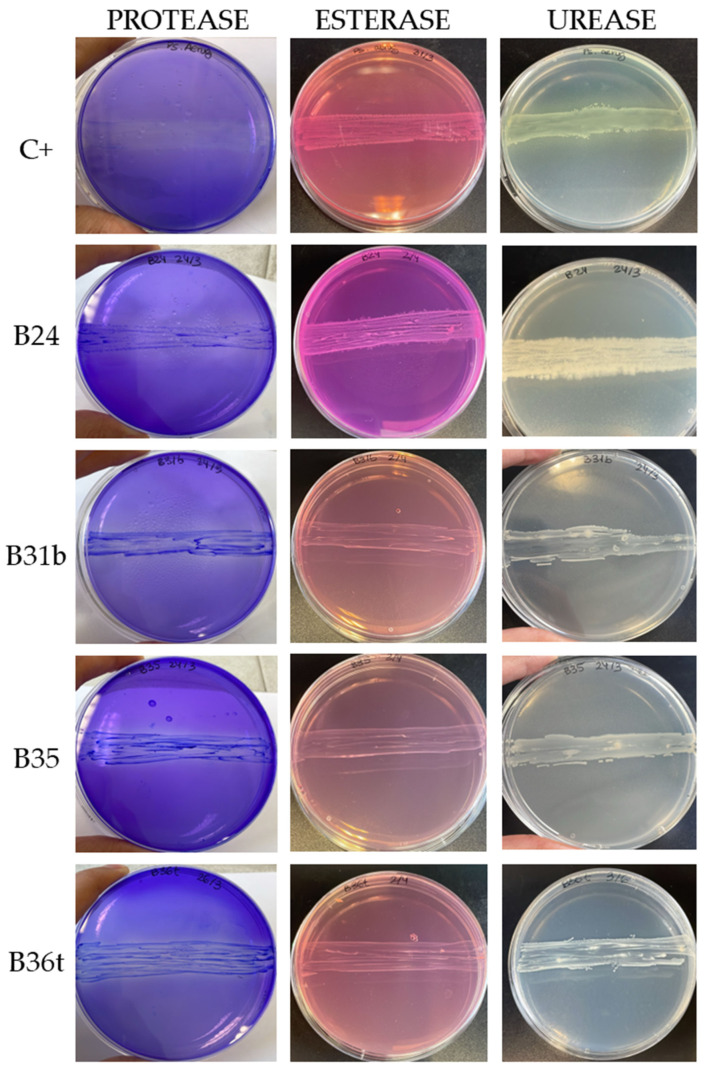
Enzymatic activity obtained using differential media: positive control C+ (*Pseudomonas aeruginosa*) and the four isolates showing positive protease, esterase, and urease activity.

As shown in Table 2, significant variability in the types and degrees of enzymes expressed by each strain was obtained. Only four isolates (B24, B31b, B35, and B36t) exhibited positive activity for all three enzymes (Figure 6). These isolates exceeded 60% DLN biodegradation in the quantitative PU biodegradation assay and belong to the *Bacillus cereus* clade (B24) and the genus *Achromobacter* (B31b, B35, and B36t).

All isolates classified within the *Bacillus cereus* clade tested positive for both protease and urease activity. However, only B24 indicates esterase activity. On the other hand, no enzymatic activity was detected in strains identified as *Aeromicrobium* sp. (B8, B13) and *Agromyces* sp. (B36a). These strains demonstrated low–moderate PU degradation in the quantitative assay, with a maximum of 36% in B36a. This suggests that other enzymes potentially involved in polyurethane degradation, such as oxidoreductases, may be active in these strains [17,40].

### 3.5. FPU Biodegradation

**Figure 7:**
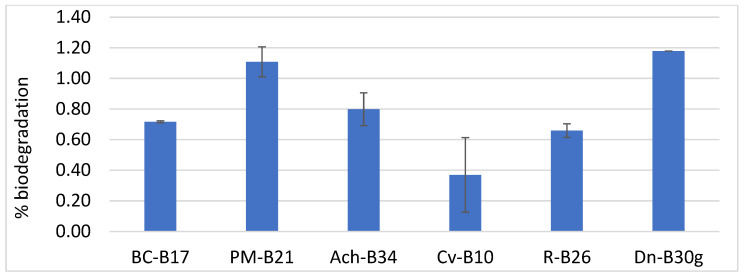
FPU biodegradation (%) obtained with selected isolates: Bc-17, *Bacillus cereus*, isolate B17; Pm, *Priestia megaterium*, isolate B21; Ach-B34, *Achromobacter* sp., isolate B34; Cv-B10, *Cupriavidus* sp., isolate B10; R-B26, *Rhodococcus erythropolis*, isolate B26; and Dn-30g, *Dermacoccus nishinomiyaensis*, isolate B30g.

For the polyether FPU biodegradation assay, only phylogenetic groups which previously exhibited biodegradation rates below 70% were selected (namely *B. cereus*, *P. megaterium*, *Achromobacter* sp., *Cupriavidus* sp., *R. erythropolis*, and *D. nishinomiyaensis*). The most efficient isolate from each group was used for testing: Bc-B17, Pm-B21, Ach-B34, Cv-B10, R-B26, and Dn-B30g. Figure 7 shows that Pm-B21 and Dn-B30g showed the highest biodegradation activity on FPU after one month of incubation, reaching up to 1.19% biodegradation in Dn-30g.

**Figure 8:**
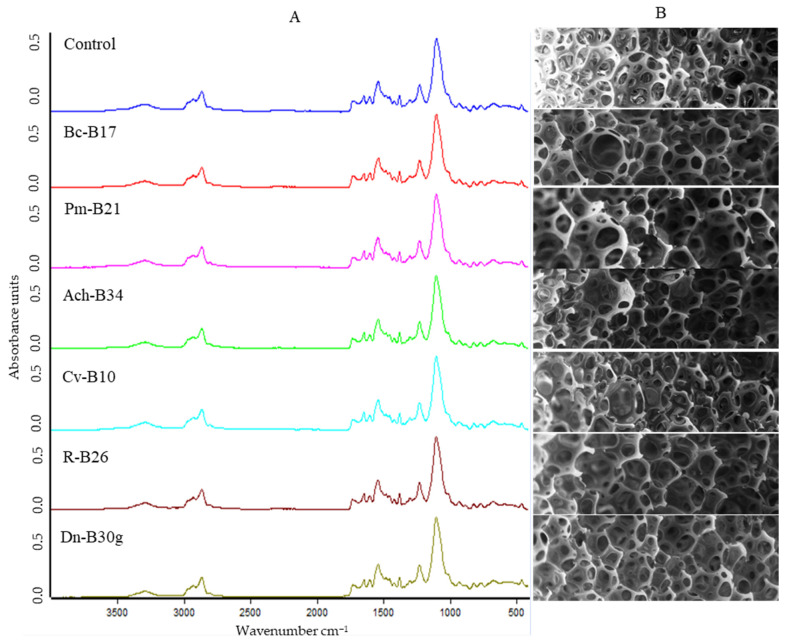
Characterization of FPU biodegradation. (**A**) FTIR spectra of FPU incubated without bacteria (control) and with selected isolates for FPU biodegradation: Bc-17, *Bacillus cereus*, isolate B17; Pm, *Priestia megaterium*, isolate B21; Ach-B34, *Achromobacter* sp., isolate B34; Cv-B10, *Cupriavidus* sp., isolate B10; R-B26, *Rhodococcus erythropolis*, isolate B26; and Dn-B30g, *Dermacoccus nishinomiyaensis*, isolate B30g. (**B**) SEM images at 25× magnification of FPU after biodegradation essay with the same isolates.

FTIR spectroscopy was used to characterize the structural changes in the FPU after the biodegradation test. The FTIR spectra were obtained as absorption versus wavenumber to evaluate changes in the functional groups of FPU after biodegradation, especially in the representative ester, urethane, and amide [41]. When comparing the spectra of the control with those of the inoculated FPU, no clear visual evidence of change is observed (Figure 8A). However, the quantitative analysis of the absolute intensity of each band relative to the control revealed decreases in specific absorption bands (Table 3), particularly at 3300, 1730, 1640, 1600, 1530, and 1220 cm^−1^, which indicates that N–H stretching, C–H stretching, C=O stretching, C=O bending, urea/urethane N–H bending, and C–N stretching vibrations were affected by microbial action. Notably, no uniform trend was observed across all isolates; instead, each strain appeared to impact different functional groups to a varying extent. In line with these results, a SEM analysis of the FPU surfaces also did not reveal clear morphological signs of degradation when compared to the control (Figure 8B), further supporting the conclusion that only minor structural modifications occurred under the tested conditions.

## 4. Discussion

The most abundant species identified in the taxonomic study belonged to the *Bacillus cereus* clade. A total of 11 strains were identified within this clade using MALDI-TOF (B1, B2, B3, B5, B9, B17, B18, B19, B20, B22, and B24), which showed similar spectral profiles, indicating a close phylogenetic relationship. Nine of these strains were isolated from TM2, one from TM1, and one from TM3. This finding is consistent with the bibliography. The genus *Bacillus* was among the first to be reported as a PU biodegrader [42,43], being the most well-known species *B. subtilis* [16]. More recent studies have also identified PU-degrading *Bacillus* strains from landfills [18,19] and established the genera as one of the predominant of PU-degrading bacteria [17].

The present study establishes the first time that *Bacillus cereus* has been identified in relation to a PU attack, with some strains (B1, B3, B17, and B20) achieving biodegradation rates of over 80%. However, their versatile metabolic profile with potential for the degradation of different pollutants has been highlighted, as they possess a varied enzymatic activity to metabolize polycyclic aromatic hydrocarbons [44], using them as a sole source of energy. Moreover, in more recent research, Jebashalomi et al. (2024) [45] have evaluated the potential of *B. cereus* in the degradation of polystyrene, and other research [46,47] have demonstrated its high potential as a biodegrader of polyethylene, another widely used polymer resistant to degradation.

Five strains belonging to the genus *Achromobacter* (B31b, B32, B34, B35, and B36t) were identified by 16S rRNA gene sequencing, as MALDI-TOF analysis did not yield conclusive results. The sequences showed high similarity (>99%) to multiple species within this genus, including *A. animicus*, *A. mucicolens*, *A. ruhlandii*, *A. deleyi*, *A. kerstersii*, *A. spanius*, *A. veterisilvae*, *A. agilis*, *A. insuavis*, *A. marplatensis*, *A. piechaudii*, *A. anxifer*, *A. pestifer*, *A. dolens*, and *A. aegrifaciens*. The close genetic proximity among these taxa prevented a definitive species-level identification.

All these strains were isolated from TM4, a landfill. Gaytán et al. (2019) [48] also reported on *Achromobacter* representation in a microbial community collected from a landfill, which can grow in a water–PU dispersion. In addition, other research has demonstrated the potential of this genus in the degradation of plastic materials. Research carried out by Das et al. (2012) [49] evidenced the biodegradative capacity of strains of the genus *Achromobacter* on different modified polyvinyl chloride (PVC) matrices under in vitro conditions. On the other hand, *Achromobacter* strains have been reported as key members of microbial consortia achieving the total biodegradation of di(2-ethylhexyl) phthalate, a widely used plasticizer that causes environmental pollution and carcinogenic, teratogenic, and mutagenic toxic effects (Bai et al., 2020) [50]. In the present study, *Achromobacter* was detected as one of the most efficient genera in DLN biodegradation, with all isolated strains belonging to this genus (B31b, B32, B34, B35, and B36t), reaching biodegradation rates above 70%.

Two strains of the genus *Aeromicrobium* (B8, B13) were also identified by partial sequencing of the 16S rRNA gene from TM2. Both showed practically identical similarity levels (98.8%) to *A. choanae* and *A. tamlense*. The low percentage of similarity obtained against other species belonging to the genus *Aeromicrobium* suggests that the isolates may represent a new species. *Aeromicrobium* was previously related to PU biodegradation. An efficient PU-degrading strain of *Aeromicrobium* was isolated by Zhang et al. (2025) [51] from a coastal mudflat, showing an up to 86% weight loss in polyester PU foam after 14 days. In the present study, the two strains identified in the *Aeromicrobium* genus exhibited a reduced polyester PU biodegradation capacity, achieving only 33.3% DLN biodegradation. In addition, Chhetri et al. (2025) [52] reported the capacity of the strain *Aeromicrobium* sp. JJY06 to biodegrade bioplastic (PCL, PBS, and PBAT) at low temperatures (25 °C), unveiling the potential of this genus for polymer degradation in cold environments.

Strain B26, identified as *Rhodococcus erythropolis* by partial sequencing of the 16S rRNA gene, was isolated from TM4 and demonstrated a high capacity to biodegrade the PU dispersion, with 89.9% of DLN biodegradation. The genus *Rhodococcus* is well known for its hydrocarbon-degrading abilities, with strains such as *Rhodococcus* sp. F92 demonstrating effective petroleum product degradation (Quek et al., 2006) [53]. Other studies have reported strains of *Rhodococcus equi* capable of degrading a model urethane molecule (Akutsu-Shigeno et al., 2006) [54] and *Rhodococcus pyridinivorans*, which can grow on solubilized PU [55]. However, this is the first study to report the activity of *Rhodococcus erythropolis* in PU biodegradation.

Strain B10 also showed a high DLN degradation rate of 90.1% and was isolated from TM2. MALDI-TOF identified it only at the genus level, although the most closely related species was *Cupriavidus respiraculi*. *Cupriavidus* (along with other genera identified in this study, *Achromobacter* and *Rhodococcus)* was detected in consortia isolated from soil and leachate samples from landfill sites using a selective method based on their growth with polyester PU powder. *Cupriavidus* abundance increased in the consortium after 50 generations of passage cultures [56]. Apart from this reference, no other reports have linked *Cupriavidus* to polyurethane biodegradation. *Cupriavidus* species are noteworthy for their metabolic versatility and ability to convert toxic compounds into polyhydroxybutyrate (PHB), a biodegradable polymer, thereby contributing both to pollution mitigation and to the circular bioeconomy. For example, *Cupriavidus necator* could transform chlorinated aromatic compounds into PHB [57]. The most remarkable feature of this genus is its ability to survive under extreme conditions, including environments with heavy metals, which makes it a promising candidate for the bioremediation of contaminated soil and water.

Among the genera identified in this study, only those previously discussed (*Bacillus*, *Achromobacter*, *Aeromicrobium*, *Rhodococcus*, and *Cupriavidus*) have been reported beforehand to exhibit PU biodegradation activity. Nevertheless, this is the first report on the isolation of the genera *Priestia*, *Dermacoccus*, *Gordonia*, *Micrococcus*, *Pseudarthrobacter*, and *Agromyces* from environmental samples directly associated with PU biodegradation. In addition, several of these bacterial genera have been described in the scientific literature as being capable of degrading other types of waste or pollutants and have demonstrated considerable potential for biotechnological and environmental applications due to their ability to synthesize high-value compounds.

*Priestia megaterium* is the second most abundant species identified in this study, with a total of five strains (B4g, B7, B16, B21, and B23) identified by MALDI-TOF, all showing high spectral similarity. All were exclusively isolated from TM2. This genus has been documented for its capacity to degrade polybutylene adipate-co-terephthalate [58] or Styrofoam [59]. In addition, *Priestia megaterium* is a well-known plant growth-promoting bacterium that has been extensively used in biotechnological processes. Its capabilities include the production of small molecules such as vitamin B12, the synthesis of biodegradable polymers like polyhydroxybutyrate (PHB), and the production of multiple proteins both in vivo and in vitro, along with whole-cell applications [60].

Two isolates were taxonomically classified as *Kocuria rhizophila* (one from TM2, one from TM4) with moderate DLN biodegradation action (over 50%). *Kocuria rhizophila* has been reported to degrade 2,4-dichloropenol [61], while other species in the genus, such as *Kocuria rosea*, are related to the biodegradation of some polluting compounds, for example, colorants [62] or naphthalene [63]. The biotechnological potential of *K. rhizophila* in novel applications, including agriculture approaches and bioremediation, is related to its heavy metal and salt resistance tolerances [64].

The remaining taxonomic groups identified in this study are represented by a unique isolate. B30g was identified from TM4 as *Dermacoccus nishinomiyaensis*. Recent findings suggest a potential role in phenol removal from the wastewater of *D. nishinomiyaensis* and a strain in the genus *Kocuria*, which may offer niche applications in environmental biotechnology [65]. *Micrococcus luteus* (B18) was detected in TM2 and has also demonstrated the ability to biodegrade various pollutant intermediates, including nitrobenzene [66] or 1-naphthol [67]. Moreover, this species can produce PHB [68], highlighting its biotechnological potential.

Strain B31c was isolated from TM4 and identified in the *Pseudarthrobacter* genus by 16S rRNA gene sequencing, with a 99.1% similarity to *P. psychrotolerans* and 99% similarity to *P. oxydans*. The genus *Pseudarthrobacter* has also been related to hydrocarbon contamination bioremediation, reporting phenanthrene biodegradation at low temperatures [69], high rates of phthalic acid degradation [70], and 2,4,6-trinitrotoluene degradation [71].

Strain B25 was identified as belonging to the *Gordonia* genus through partial 16S rRNA gene sequencing. *Gordonia* species, particularly *G. polyisoprenivorans*, have been associated with the biodegradation of polyethylene due to their biofilm-forming capabilities [72]. *Gordonia* is also known for its ability to degrade a broad spectrum of recalcitrant compounds, including alkanes, sulfur-containing molecules, and hydrocarbons [73]. B25 showed a high identity with several *Gordonia* species, including *G. effusa* and *G. terrae* (98.8%), as well as *G. hongkongensis* (98.3%). Similarly to the situation with *Aeromicrobium*, similar values below 99% suggest that B25 may represent a new species.

As with the previous case involving *Achromobacter* strains, accurate species-level identification was impossible due to the high phylogenetic similarity. These results highlight the limitations of MALDI-TOF and partial 16S rRNA gene sequencing for species-level identification within certain phylogenetically close bacterial groups, as these methods cannot fully capture the genomic complexity of bacteria. Consequently, there is a need to complement these techniques with more resolutive and accurate genomic analyses, such as whole-genome sequencing [74].

Protease activity was the most prevalent among the tested isolates and was detected in 27 of the 33 strains. This was followed by urease activity, observed in 24 strains, while esterase activity was detected in only 11 strains. These findings contrast with previous reports, in which the esterase activity had been most frequently associated with PU biodegradation [14,23,48,56].

No clear relationship was observed between the expression of the evaluated enzymes and either the taxonomic classification of the isolates or their DLN biodegradation efficiency. Among the most effective taxonomic groups identified in this study, there were *B. cereus* with isolated B1 (90.1%), *P. megaterium* with B21 (88.7%), *Achromobacter* with B34 (90.2%), *Cupriavidus* sp. With strain B10 (90.1%), and *R. erythropolis* with strain B26 (89.8%). However, none of these strains simultaneously exhibited the three enzymatic activities studied, and most did not show particularly high levels of enzymatic expression. The only exception was *P. megaterium* B21, which exhibited high protease and urease activity.

To the best of our knowledge, the enzymatic activity of *P. megaterium* in the context of plastic degradation is scarcely documented in the current literature. In our study, *B. cereus* B1 showed medium protease activity and low urease activity. A general tendency for protease production was observed among *B cereus* strains, with 10 out of 11 isolates exhibiting high or medium protease activity. Previous studies have highlighted the genus *Bacillus* as a prolific source of proteases, which often remain stable across a wide range of pH and temperatures and are thus extensively utilized in industrial applications such as detergents, food processing, pharmaceuticals, and textiles [75].

*Achromobacter* sp. B34 displayed low urease and medium esterase activity. The role of esterases in plastic degradation by *Achromobacter* sp. has been previously described [76,77], supporting the potential relevance of this enzymatic route in PU biodegradation. In contrast, *Cupriavidus* sp. B10 showed only medium protease activity. However, Su et al. (2023) [56] reported protease and urease activity in microbial consortia that contain *Cupriavidus* strains; no direct evidence has been found linking this genus to esterase or other relevant enzymatic activities involved in PU degradation.

*R. erythropolis* B26 showed low protease activity and high urease activity. The *Rhodococcus* is known to possess a broad enzymatic arsenal capable of degrading various plastic polymers, including PET and PU. Orts et al. (2025) [55] reported significant activity of cutinases and esterases in *Rhodococcus pyridinivorans* during the degradation of solubilized PU, reinforcing the enzymatic versatility of this genus.

Microbial PU degradation can proceed through multiple enzymatic pathways. A single microorganism may produce several enzymes that act synergistically, or degradation may occur via less commonly studied enzymes such as urethanases, amidases, oxidoreductases, cutinases, and hydrolases [7,78]. This enzymatic diversity might explain the lack of correlation between the three tested enzymes and the biodegradation efficiency of the isolates.

It should be emphasized that, although DLN has been widely employed as a model substrate to evaluate polyurethane biodegradation [15,16,19,24,35,51,79,80], its exact composition and molecular structure are not publicly disclosed, since it is a commercial and registered product. Previous research has suggested that DLN contains both urethane and ester linkages and proposed tentative structures based on polyester-polyurethane backbones [79,80]. However, additional analyses of cleavage products released after biodegradation indicate that the polymer matrix may be more complex than initially assumed [24,79]. Consequently, the transparency loss and weight reduction observed in this study should be interpreted as evidence of overall DLN degradation rather than as a direct demonstration of urethane bond cleavage. This limitation must be considered when extrapolating results to more defined PU substrates. Nonetheless, the use of DLN remains valuable for screening microbial PU-degrading activity, as it allows comparative analyses across studies and has been validated as a proxy for assessing potential biodegraders [15,18,24,34,51]. Complementary studies on well-characterized polyurethanes are required to elucidate specific enzymatic mechanisms.

To conclude this study, the most effective strains in the DLN biodegradation assay were tested for their potential to degrade other PU formats, specifically polyether FPUs, which are considered among the most recalcitrant PU materials [4,7,15]. It is well established that polyether PU bonds are more susceptible to degradation via physicochemical oxidation [81]. In contrast, the enzymatic oxidation of ether bonds has not been demonstrated to date [24]. Consequently, ether bonds in polyether FPU appear to be resistant to microbial attack, and the biodegradation of these materials is thought to occur primarily through enzymatic hydrolysis of urethane and urea linkages [82]. However, this degradation pathway results in much slower biodegradation rates when compared to a polyester-based FPU, as corroborated by the findings of the present study, in which the 1.19% degradation of polyether FPU was observed. In addition, DLN is an aqueous PU dispersion that provides a highly accessible substrate for microbial enzymes [7,79]. In contrast, FPUs are thermoset, crosslinked materials with a far more complex molecular architecture, reduced surface accessibility, and higher structural recalcitrance [3,4,7]. Our results are consistent with existing publications, where most reports on polyether FPU biodegradation involve fungal species [24,82,83]. Studies demonstrating bacterial degradation of polyether PU are very limited. Marova et al. (2007) [84] reported the partial biodegradation of FPU that incorporates different cellulose, replacing commercial polyether polyol by strains *Arthorobacter globiformis*, *Comamonas acidovorans*, *Bacillus* sp., and *Thermus* sp. Kemona and Piotrowska (2016) [85] found that *Staphylococcus xylosus* and *Rhodococcus* spp. exhibited significant growth in a medium containing polyether PU as the sole carbon source. Given the resistance of ether bonds to enzymatic cleavage, future research should explore strategies that combine abiotic oxidation (e.g., through UV or chemical treatment) with subsequent microbial degradation, thereby enhancing the biodegradability of polyether-based PU foams.

Despite the enzymatic screening carried out in this work, several limitations must be acknowledged. As observed in the FTIR analyses, each bacterial isolate appeared to affect different functional groups of PU, suggesting that a wide variety of enzymatic activities may be involved in PU biodegradation [4,7,78]. Furthermore, the characterization techniques employed in this study (FTIR and SEM) did not reveal clear structural or morphological changes in the polymer, which is likely attributable to the very low percentage of FPU biodegradation observed. Similar results, where only slight decreases in band intensities are detected, have also been reported in other studies of PU biodegradation [34,56], indicating that these subtle changes are typical when degradation rates are low. These methodological constraints limited our ability to fully elucidate the chemical or structural modifications induced by microbial activity. Therefore, future studies should incorporate broader enzymatic assays and advanced approaches such as proteomics, metabolomics, or high-resolution surface characterization to better understand the enzymatic diversity and molecular mechanisms driving PU biodegradation.

Other important aspects for future work on PU biodegradation include the use of microbial consortia. Several studies [50,56] have demonstrated that PU decomposition is more efficient when different bacterial species collaborate within consortia than when they act individually. These consortia target specific PU groups, causing their degradation. They are also more resistant to environmental variations and can degrade a wider variety of complex polymers. Additionally, determining the optimal environmental conditions for PU biodegradation would also be relevant, since enzymatic activity is influenced by factors such as temperature and pH. Understanding these parameters would allow the process to be optimized for each bacterial strain.

## 5. Conclusions

This study provides a comprehensive characterization of PU-degrading microorganisms isolated from industrial and environmental sites. The results highlight both the taxonomic diversity and the phenotypic potential of selected strains, particularly their ability to degrade Impranil DLN, a colloidal PU dispersion commonly used as a model substrate. To the best of our knowledge, this is the first report identifying the genera *Priesta*, *Dermacoccus*, *Gordonia*, *Micrococcus*, *Pseudarthrobacter*, and *Agromyces* as PU-degrading microorganisms.

A positive correlation was observed between DLN biodegradation and activity against polyether-based FPU, although no pronounced structural and morphological changes were detected with FTIR and SEM analyses, consistent with the low degradation percentages achieved. These findings reinforce both the technical challenges of detecting early-stage PU biodegradation and the need for more sensitive analytical approaches to capture the molecular-level transformations induced by microbial activity. These low biodegradation rates obtained for polyether-based FPUs reaffirm their recalcitrant nature compared to polyester-based PUs and emphasize the need for integrated approaches combining abiotic pretreatments, such as photo-oxidation, with microbial action, as polyether bonds are more susceptible to physicochemical than enzymatic attacks.

Conversely, no clear correlation was observed between the expression levels of the tested enzymatic activities (protease, urease, and esterase) and the degradation capacity of the strains. This suggests that PU biodegradation may be driven by alternative enzymatic systems that were not assessed in this study. Enzymes such as cutinases, amidases, oxidoreductases, and other hydrolases have been previously associated with PU degradation and could play a more relevant role in these microbial processes.

The diverse phylogenetic affiliations of the isolates underscore the complex and multifactorial nature of microbial PU degradation. The coexistence of well-documented degraders, such as *Bacillus*, with novel candidates highlights the opportunity to discover new metabolic routes and enzymatic mechanisms. Future studies focusing on whole-genome sequencing, proteomics, and metabolic profiling will be essential to elucidate the molecular basis of PU biodegradation and to harness these microorganisms for environmental biotechnological applications. The insights gained from these strains could be leveraged to develop optimized microbial consortia or recombinant enzyme systems for the treatment of PU-containing waste streams.

Overall, the isolates identified in this study represent a promising biological resource for environmental biotechnology strategies to mitigate plastic and xenobiotic pollution.

## Figures and Tables

**Table 1:** Impranil DLN W-50 (DLN) biodegradation (%) obtained with the isolates, grouped by their origin: TM1, the PU foam by-product disposal area of an upholstery factory; TM2, the PU foam by-product disposal area in a mattress factory; TM3, the surrounding area of a former upholstery factory; and TM4, a local landfill.

Isolate	Origin ^1^	% Biodegradation ^2^
B1	TM1 ^a^	90.1 ± 5.4
B2	TM2 ^c^	7.0 ± 6.7
B3		82.7 ± 1.0
B4g		54.2 ± 7.0
B5		38.8 ± 3.8
B8		12.9 ± 3.0
B9		47.2 ± 8.5
B10		90.1 ± 9.9
B13		33.3 ±8.9
B15		51.4 ± 8.9
B16		32.8 ± 9.9
B17		86.6 ± 9.6
B18		38.1 ± 3.1
B19		46.7± 3.8
B20		84.8 ± 9.0
B21		88.7 ± 2.4
B22		4.7 ± 2.3
B23		82.6 ± 2.9
B24	TM3 ^b,c^	64.0 ± 9.7
B25		44.3 ± 7.2
B26	TM4 ^a,b^	89.8 ± 1.6
B30p		47.0 ± 10.6
B30g		72.6 ± 9.2
B31b		81.1 ± 6.0
B31c		79.7 ± 9.4
B32		74.8 ± 10.1
B34		90.2 ± 4.1
B35		70.1 ± 9.0
B36t		81.7 ± 8.6
B36a		36.0 ± 2.6

^1^ The origin labeled with different superscripts (a, b, or c) showed significant differences in the average biodegradation percentages (*p* > 0.05). ^2^ The values were an average of three repetitions ± standard deviation.

**Table 2:** Enzymatic activity is determined by growth in three differential media. +++: high activity; ++: medium activity; +: low activity; and -: no activity.

Isolate	Identification	Protease	Urease	Esterase
B1	*Bacillus cereus*	++	+	-
B2		+++	++	-
B3		+++	++	-
B5		+++	+	-
B9		+++	++	-
B17		++	+	-
B19		+++	++	-
B20		++	+	-
B22		+++	+	-
B24		+	+++	+
B4g	*Priestia megaterium*	+	+++	-
B7		+++	+++	-
B16		++	+++	-
B21		+++	+++	-
B23		-	++	+++
B8	*Aeromicrobium* sp.	-	-	-
B13		-	-	-
B31b	*Achromobacter* sp.	+	++	+
B32		-	-	++
B34		-	+	++
B35		+	+	+
B36t		+	+	+
B15	*Kocuria rhizophila*	++	-	-
B30p		++	-	+++
B10o	*Cupriavidus* sp.	++	-	-
B18	*Micrococcus luteus*	++	++	-
B25	*Gordonia* sp.	+	+++	-
B26	*Rhodococcus erythropolis*	+	+++	-
B30g	*Dermacoccus nishinomiyaensis*	+++	-	+++
B31c	*Pseudoarthrobacter* sp.	+	++	-
B36a	*Agromyces* sp.	-	-	-

**Table 3:** Decrease in the intensity of absorption bands of functional groups, calculated relative to the control, after FPU biodegradation with selected isolates: Bc-17, *Bacillus cereus*, isolate B17; Pm, *Priestia megaterium*, isolate B21; Am; Ach, *Achromobacter* sp., isolate B34; Cv, *Cupriavidus* sp., isolate B10; R, *Rhodococcus erythropolis*, isolate B26; and Dn, *Dermacoccus nishinomiyaensis*, isolate B30g.

Functional Group	Typical Assignment	Wave Number (cm^−1^)	BC- B17	PM- B21	Ach- B34	Cv- B10	R- B26	Dn- B30g
O–H/N–H	Stretching vibrations (hydrogen bonded OH, NH, or urethane)	3300	14.8%	13.1%	14.8%	6.6%	14.8%	13.1%
C–H	Aliphatic C–H stretching	2860	1.2%	2.5%	1.2%	2.5%	1.2%	1.2%
C=O	Free urethane carbonyl (non-hydrogen bonded)	1730	0.0%	7.1%	3.5%	4.7%	4.7%	0.0%
C=O	Hydrogen-bonded carbonyl (urethane or ester)	1640	14.2%	1.6%	0.8%	0.8%	0.0%	4.7%
C=C/aromatic	Aromatic ring stretching (from isocyanate-derived segments)	1600	2.6%	6.0%	5.2%	3.4%	5.2%	2.6%
N–H bending	Amide II band (NH bending coupled with C–N stretching)	1530	2.9%	4.6%	3.3%	3.3%	2.9%	3.3%
C–N stretching/ N–H bending	Amide III band (urethane linkage)	1220	0.9%	3.7%	0.9%	1.9%	1.4%	1.4%
C–O–C (ether)	Stretching vibration of polyether segment	1100	0.3%	2.6%	0.0%	1.6%	0.3%	0.5%
C–H bending	Out-of-plane bending (substituted aromatic rings or ether-related modes)	930	4.5%	13.6%	3.0%	3.0%	4.5%	4.5%

## Data Availability

The data presented in this study are available on request from the corresponding author. The data are not publicly available due to the absence of a suitable public repository for experimental datasets of this nature.
